# The Therapeutic Potential of Plant Polysaccharides in Metabolic Diseases

**DOI:** 10.3390/ph15111329

**Published:** 2022-10-27

**Authors:** Xiao-Fang Wang, Xue Chen, Yong Tang, Jian-Ming Wu, Da-Lian Qin, Lu Yu, Chong-Lin Yu, Xiao-Gang Zhou, An-Guo Wu

**Affiliations:** 1 Sichuan Key Medical Laboratory of New Drug Discovery and Druggability Evaluation, Luzhou Key Laboratory of Activity Screening and Druggability Evaluation for Chinese Materia Medica, School of Pharmacy, Education Ministry Key Laboratory of Medical Electrophysiology, Southwest Medical University, Luzhou 646000, China; 2 State Key Laboratory of Quality Research in Chinese Medicine, Macau University of Science and Technology, Macau 999078, China; 3 Hunan Key Laboratory of the Research and Development of Novel Pharmaceutical Preparations, College of Pharmacy, Changsha Medical University, Changsha 410219, China

**Keywords:** plant polysaccharides, metabolic disease, characteristics, pharmacological activities, mechanism of action

## Abstract

Plant polysaccharides (PPS) composed of more than 10 monosaccharides show high safety and various pharmacological activities, including immunoregulatory, antitumor, antioxidative, antiaging, and other effects. In recent years, emerging evidence has indicated that many PPS are beneficial for metabolic diseases, such as cardiovascular disease (CVD), diabetes, obesity, and neurological diseases, which are usually caused by the metabolic disorder of fat, sugar, and protein. In this review, we introduce the common characteristics and functional activity of many representative PPS, emphasize the common risks and molecular mechanism of metabolic diseases, and discuss the pharmacological activity and mechanism of action of representative PPS obtained from plants including *Aloe vera*, *Angelica sinensis*, pumpkin, *Lycium barbarum*, *Ginseng*, *Schisandra chinensis*, *Dioscorea pposite*, *Poria cocos*, and tea in metabolic diseases. Finally, this review will provide directions and a reference for future research and for the development of PPS into potential drugs for the treatment of metabolic diseases.

## 1. Introduction

With the successful treatment of many infectious diseases worldwide, noncommunicable diseases (NCDs) have become a major risk factor for human health and life. Of all these NCDs, metabolic syndrome (MetS) affecting metabolic health poses the largest global threat [1]. Metabolic diseases are caused by many risk factors, including obesity, hypertension, insulin resistance (IR), genetics, and the environment. Among them, obesity has become the most common and important in view of statistics that indicate that nearly half of the diabetes burden and one-quarter of the heart disease burden are reportedly caused by being overweight or obese [2]. Emerging evidence indicates that most common diseases including diabetes, CVD, nonalcoholic fatty liver disease (NAFLD), and central nervous system (CNS)-related disease are metabolic diseases [3].

Currently, with the improvement in quality of life, an increasing number of people tend to choose natural medicines, especially plant-based supplements, for the prevention and treatment of diseases [4,5]. Polysaccharides are a kind of component that can be widely found in different plant species, and the structures comprise a variety of monosaccharides linked by glycoside bonds. Numerous studies indicate that plant polysaccharides (PPS) show multiple functional activities, mainly including immunity regulation and antitumor, anti-inflammatory, antivirus, antiradiation, and hypoglycemic effects. Most importantly, in vivo experiments have demonstrated that most PPS are relatively nontoxic and have few side effects [6]. Recently, many PPS have been widely studied in the potential treatment of metabolic diseases. For example, *Aloe* polysaccharides (APs) improve diabetes by activating the PERK and IRE1 pathways [7]. *Angelica sinensis* polysaccharides(ASPs) exert a neuroprotective effect in Alzheimer’s disease (AD) by regulating the Aβ25-35 neurotransmitter balance, free radiation metabolism, inflammation, and neuronal apoptosis [8]. In addition, *Poria cocos* polysaccharides (PCPs) improve atherosclerosis (AS) by regulating blood lipid levels, decreasing malondialdehyde (MDA) levels, and increasing superoxide dismutase (SOD) activity [9]. Although many kinds of PPS have shown positive effects in lowering blood sugar and blood pressure and protecting the liver and heart [10], there are still problems, such as poor targeting, poor stability, rapid blood clearance rate, and unconcentrated scope of action, which seriously affect bioavailability and clinical application [11,12]. In this review, we introduce the common characteristics and functional activity of PPS. In addition, the common risks and molecular mechanism of metabolic diseases are emphasized, and the pharmacological activity and mechanism of action of several representative PPS in metabolic diseases are summarized and discussed. Finally, this review should provide directions for future research of PPS in metabolic diseases.

## 2. Plant Polysaccharides 

### 2.1. Type

Phytochemical studies reveal that polysaccharides are the most abundant components in plants [13]. To date, many types of PPS, including starch, cellulose, polysaccharide, and pectin, have been reported. Of these, starch and cellulose are the two main groups in plants. The polymers have been found to form cell walls, which are also known as cell wall polysaccharides such as cellulose. In addition, other polymers are the main source of energy and water in many plant organs and are called storage polysaccharides, and they include starch [14]. According to the different parts of plants, PPS can also be divided into polysaccharides of plant stem, leaf, flower, fruit, and root [15].

### 2.2. Composition

Recent studies have found that PPS are an important class of biological polymers composed of more than 10 monosaccharides and are linked by glycosidic bonds, and the molecular weight is up to tens of thousands or even millions [16]. As shown in Table 1, although there are some differences in the composition of polysaccharides in different plants, the main monosaccharides are glucose, fructose, galactose, arabinose, xylose, rhamnose, fucose, mannose, and uronic acid. For example, acemannan in *Aloe vera* is mainly composed of acetylated Man, Glc, and Gal [17]. ASPs in *Angelica sinensis* are mainly composed of glucuronic acid, glucose, arabinose, and galactose [17,18]. The novel natural low-molecular-mass polysaccharide (SLWPP-3) in pumpkin (*Cucurbita moschata* Duch.) mainly comprises rhamnose, glucose, arabinose, galactose, and uronic acid [19].

### 2.3. Structure

**Figure 1:**
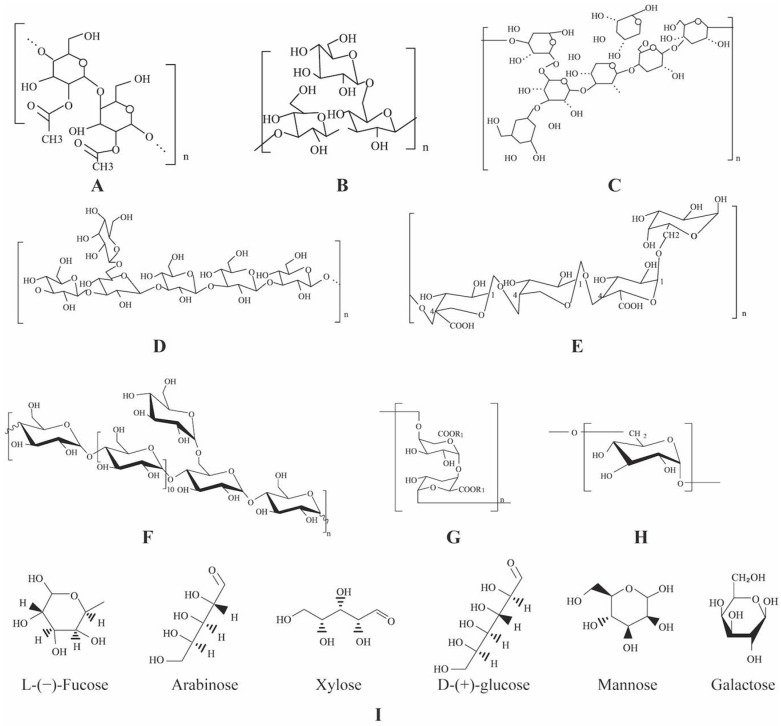
Chemical structures of representative PPS. (**A**) *Aloe vera,* (**B**) *Angelica sinensis,* (**C**) *Schisandra chinensi,* (**D**) *Poria cocos,* (**E**) *Panax ginseng*, (**F**) Pumpkin (*Cucurbita moschata*), (**G**) Tea (*Camellia sinensis*), (**H**) *Dioscorea opposita*, (**I**) *Lycium barbarum*.

The structure of PPS comprises monosaccharides linked by glycosidic bonds. The glycosidic bonds of PPS are mainly α-(1→ 6)-D, α-(1→ 4)-D, and β-(1→ 4)-D [6]. Most importantly, the biological activities of polysaccharides are closely related to their primary and higher structures [20]. However, due to large molecular weight (up to 10 million), complicated structure, and the absence of model compounds, few studies have reported the structure–activity relationships of PPS [16]. Structurally, polysaccharides can be divided into primary, secondary, tertiary, and quaternary structures, which is more complicated than the structure of proteins and DNA [21]. The higher structure of polysaccharides is a complex high-order structure formed by noncovalent bond interactions of side chains based on the primary structure [22]. In addition, many functional groups of PPS, such as phosphoric acid groups, sulfuric acid groups, and methylated groups, connect to sugar groups and exert pharmacological effects [23]. The chemical structures of several important PPS, that is *Aloe vera* [24], *Angelica sinensis* [25], *Schisandra chinensis* [26], *Poria cocos* [27], and *Panax ginseng* [28], Pumpkin (*Cucurbita moschata*) [29], Tea (*Camellia sinensis*) [30], *Dioscorea opposita* [31], and *Lycium barbarum* [32], are shown in Figure 1.

### 2.4. Functional Activity

It was found that the vast majority of PPS are relatively nontoxic and do not cause significant side effects [6]. Studies have demonstrated that PPS have various functional effects, such as immunomodulatory, antioxidative, antitumor, and antidiabetic activity [33]. The functional activities of several representative PPS are summarized in Table 1. For example, acemannan, an important bioactive polysaccharide in *Aloe vera* [24], was reported to have the potential of prebiotics [34]. The high-molecular-weight components of acemannan could be degraded by intestinal microbiota to form oligosaccharides that inhibit intestinal glucose absorption, thereby lowering blood glucose [24]. Acemannan was also found to reduce hepatic fat accumulation [17] and promote bone growth [17]. *Angelica sinensis* polysaccharide (ASPs) has immunological [35], hypoglycemic [36], and liver-protective effects [37]. *Panax ginseng* polysaccharides (GPs) have hypoglycemic, blood pressure-lowering, and antidepression effects [38]. Therefore, PPS are the important bioactive components that are attracting increasing attention from researchers.

**Table 1:** Composition, molecular weight, extraction method, and functional activity of representative polysaccharides from different plant parts.

PPS	Plant Parts	Monosaccharides	MW (kDa)	Extract Methods	Functional Activities	Reference
APs	Stems and leaf	Glucose, mannose, galactose, arabinose, xylose	200–523	HWE, ETE	Regulating immunity, lowering blood glucose, inhibiting tumor progression, reducing inflammation, improving oral disease, regulating CVDs, promoting bone growth	[17,39,40,41,42,43,44]
ASPs	Rhizome	Glucose, mannose, galactose, rhamnose, arabinose, xylose	5.1–2300	HWE	Regulating immunity, inhibiting tumor progression, reducing radiation, improving hematopoiesis, lowering blood sugar and blood lipids, protecting the liver, inhibiting oxidative damage and protecting nerves, reducing joint inflammation	[35,36,37,45,46,47,48,49]
Pumpkin (*Cucurbita moschata*) polysaccharides (PPs)	Fruit	Galactose, glucose, arabinose, xylose, glucuronic acid	-	ALE, UAE, HWE	Inhibiting cancer progression, reducing oxidation, lowering blood sugar, reducing bacteria, reducing toxicity, reducing blood pressure, reducing blood lipids, lowering cholesterol levels, assisting the healing process in wounds	[19,50,51,52,53,54,55]
*Lycium barbarum* polysaccharides (LCPs)	Rhizome	Rhamnose, fucose, arabinose, galactose	10–2300	UAE, EAM, MAM, SFM	Enhancing the intestinal microbiota, boosting beneficial bacteria levels, modulating innate immune response, reducing oxidation, delaying aging, increasing metabolism lowing intraocular pressure, regulating immunity, inhibiting tumor progression, improving neurological diseases, lowering blood sugar	[56,57,58,59,60]
GPs	Rhizome	Arabinose, galactose, rhamnose, galacturonic acid, glucuronic acid	3.2–1900	ETE	Relieving depression, reducing blood glucose, regulating immunity, inhibiting cancer progression, reducing oxidation, reducing radiation	[38,61,62]
*Schisandra chinensis* polysaccharides (SCPs)	Fruit	Rhamnose, fucose, arabinose, xylose, mannose, glucose, galactose	-	ETE, HWE	Lowering blood sugar, relieving fatigue, relieving a cough, reducing inflammation, improving neurological diseases, reducing hyperprolactinemia, promoting regeneration, reversing liver injury, inhibiting cancer progression, protecting the intestines	[10,63,64,65,66,67,68,69]
*Dioscorea opposita* polysaccharides (DOPs)	Rhizome	Glucose, mannose, xylose, galactose, arabinose, fucose	-	HWE, ETE	Reducing blood sugar, inhibiting cancer progression, reducing oxidation, promoting endometrial epithelial proliferation, regulating immunity, protecting the heart	[31,70,71,72]
PCPs	Rhizome	Glucose, fucose, arabinose, xylose, mannose, galactose	41–500	HWE, MAE, EE, UE	Reducing liver injury, inhibiting cancer progression, reducing inflammatory factors and blood lipid levels, relieving depression, regulating immunity	[27,73,74,75,76,77,78]
Tea (*Camellia sinensis*) polysaccharides (TPs)	Rhizome	Glucose, rhamnose, arabinose, mannose, ribose, xylose, galactose, fucose, galacturonic acid	1000–5000	HWE, MAE, EE, UAE	Inhibiting cancer, reducing blood sugar, reducing oxidation, reducing inflammatory factors and blood lipid levels, relieving fatigue	[79,80]

MW, molecular weight; AHE, acid hydrolysis extraction; ALE, alkaline; UAE, ultrasound-assisted enzymatic; HWE, hot water extraction; EAM, enzyme-assisted extraction method; MAM, microwave-assisted extraction method; SFM, supercritical fluid extraction; ETE, ethanol extraction; MAE, microwave-assisted extraction; EE, enzymatic extraction; UE, ultrasound extraction; EE, enzymatic extraction.

## 3. Metabolic Diseases

Metabolic diseases are characterized by disorder of the generation and storage of energy. In general, the substances, including sugar, protein, fat, vitamins, and minerals, in the human body cannot be metabolized effectively, resulting in the occurrence and development of metabolic diseases, such as obesity, diabetes, CVD, nonalcoholic steatohepatitis (NASH), nervous system disease (NSDs), and cancer. These metabolic diseases are thought to be affected by a complex interplay between genetics, epigenetics, susceptibility, environmental factors, and nutrition [81].

### 3.1. Common Risk Factors

**Figure 2:**
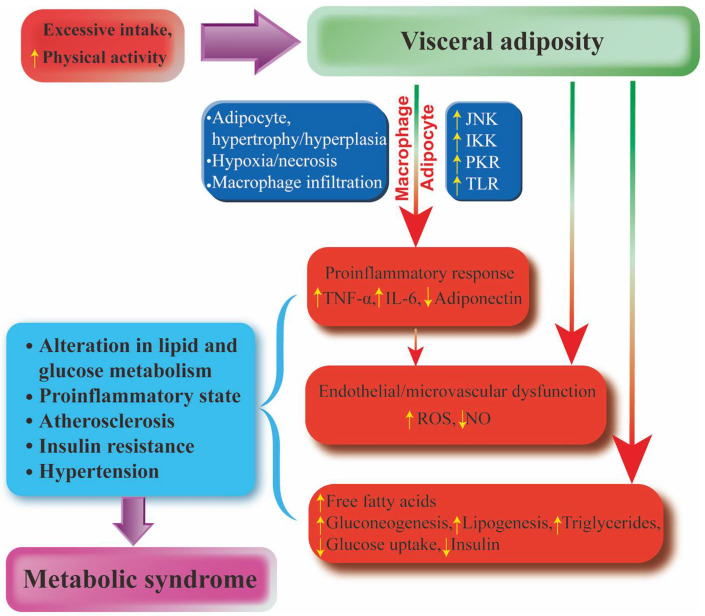
The association between abdominal obesity and MetS. Excessive intake and reduced physical activity can lead to abdominal obesity. On the one hand, abdominal obesity leads to increased TNF-α, IL-6, and ROS and decreased adiponectin and nitric oxide through inflammatory pathways. On the other hand, an increase in FFA leads to gluconeogenesis, which increases fat and TG production and reduces glucose intake and insulin production. This leads to a series of metabolic diseases.

MetS is a progressive, interdependent pathophysiological state consisting of a number of causal risk factors that become increasingly resistant to illness. Inflammation, visceral obesity, ectopic (especially liver and muscle), IR, and sugar consumption play key roles in disease pathogenesis [82]. To better reflect the underlying pathophysiology of MetS, inflammatory and prethrombotic markers, including insulin levels, plasminogen activator inhibitors, C-reactive protein (CRP), interleukin-6 (IL-6), uric acid (UA) levels, and microalbuminuria (MUA) are considered. In addition, phenotypic features, such as chronic kidney disease (CKD) and NAFLD, polycystic ovary syndrome (PCOS), and obstructive sleep apnea (OSA) are highlighted [83]. As reported by Kassi et al., other abnormalities such as chronic proinflammatory, prethrombotic states, and sleep apnea have also been added to the entity of the syndrome, making the definition of MetS more and more complete [83]. At the same time, they also revealed that there is some debate as to whether this entity is a substitute for comprehensive risk factors that expose individuals to specific risks [81]. Emerging evidence indicates that obesity, abdominal adiposity, or indicators of IR, impaired glucose metabolism, hypertension, and atherogenic dyslipidemia are common risk factors for various metabolic diseases [84]. In general, MetS is defined as a combination of three or more risk factors, including abdominal obesity, high triglycerides (TG), low- and high-density lipoprotein cholesterol (LDL-C and HDL-C), and high blood pressure, according to the consensus statement of the National Heart, Lung and Blood Institute and the American Heart Association [85]. Visceral obesity has been shown to be a major trigger of MetS, thus underscoring the importance of a high-calorie diet and lack of exercise as major causative factors [84]. Among all proposed mechanisms, alterations in lipid and glucose metabolism, IR, chronic inflammation, hypertension, etc., appear to be responsible for the initiation of MetS. Obesity is associated with MetS primarily through inflammatory processes [86]. Adipose tissue produces and releases a variety of pro- and anti-inflammatory factors, including the adipokines leptin, adiponectin, and resistin, as well as cytokines and chemokines, such as tumor necrosis factor-α (TNF-α), leptin, IL-6, and monocyte chemoattractant protein-1 (MCP-1) [87]. Among them, IL-6 strongly stimulated hepatocytes to produce and secrete CPR, indicating the existence of a proinflammatory state [88]. Furthermore, the accumulation of free fatty acids (FFAs) in obesity activates a cascade of proinflammatory serine kinases, such as IkB kinase and c-Jun-terminal kinase (JNK), which, in turn, promotes the release of IL-6 from adipose tissue, triggering the synthesis and secretion of CPR by hepatocytes [89]. This leads to metabolic disorders, such as IR, lipotoxicity, and changes in glucose metabolism and AS. IR increases the production of renin-angiotensin II, resulting in the production of reactive oxygen species (ROS). In turn, oxidative stress can lead to endothelial nitric oxide synthase (eNOS) imbalance and vascular endothelial dysfunction. Together with ROS involved in inducing mitochondrial dysfunction and macromolecular damage, oxidative stress is involved in the pathogenesis and progression of CVD, such as AS, hypertension heart failure, and peripheral arterial diseases [90]. Moreover, the IR-mediated increase in circulating FFAs is thought to play a key role in the pathogenesis of MetS. The increased FFAs promote the protein kinase activation in the liver, thereby promoting gluconeogenesis and lipogenesis. Eventually, compensation fails and insulin secretion decreases, thereby increasing the risk of CVD [91] (Figure 2).

### 3.2. Cardiovascular Disease

**Figure 3:**
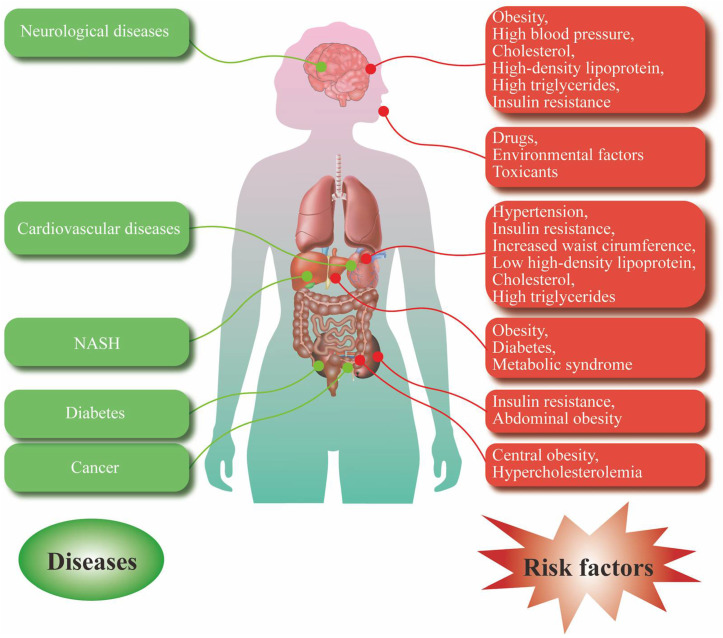
Common risk factors and various metabolic diseases. Risk factors for neurological disorders include obesity, high blood pressure, HDL-C, and high TG and IR. Risk factors for CVD include hypertension IR, increased waist circumference, low HDL, HDL-C, and high TG. Risk factors for NASH include obesity, diabetes, and MetS. Risk factors for diabetes include IR and abdominal obesity. Risk factors for cancer include central obesity and hypercholesterolemia. At the same time, drugs and environmental toxins can also affect human health from the respiratory tract and cause a series of metabolic diseases.

CVD is the general term for cardiovascular and cerebrovascular diseases, including systemic vascular disease or systemic vascular disease in the heart and brain. It mainly includes AS, aneurysm disease, coronary heart disease, cerebral infarction, and hypertension [92]. Numerous studies have shown that the occurrence of CVD is closely related to metabolic disorder [93], which manifests as hyperinsulinemia (HINS), hypertension, elevated LDL, and IR [94]. For example, IR leads to vascular stiffness, dysfunction of endothelial vessels, and vascular smooth muscle, which finally develop into various CVDs, such as AS, hypertension, coronary heart disease, and stroke [95]. In addition, LDL in plasma cholesterol increases the deposition of lipids on the arterial wall, resulting in coronary heart disease and AS [96] (Figure 3). 

### 3.3. Type 2 Diabetes Mellitus 

Type 2 diabetes mellitus (T2DM) is a group of MetS that is characterized by absolute or relative insufficiency of insulin secretion, and decreased sensitivity of target organs to insulin, followed by metabolic disorders of fat, protein, water, and electrolytes [97]. In recent years, a large number of studies have shown that obesity, genetics, islet dysfunction, and intestinal flora are involved in the process of energy metabolism, which is closely related to the occurrence and development of T2DM [97]. IR is an important metabolic risk factor in T2DM. During the initial stage of IR, β cells in the pancreas secrete insulin to control blood sugar, leading to HINS in these individuals. However, when individuals are unable to maintain the levels of normal blood sugar through this compensatory mechanism, they develop T2DM [98]. In addition, obesity alone, especially abdominal adiposity, is a major determinant of the development of T2DM [99]. Furthermore, Larsen et al. first reported that there were significant differences in gut microbiota between T2DM patients and normal population [98]. Intestinal flora are also an important factor in the occurrence and development of T2DM [97]. In an established animal model of T2DM, liver fat was found to be supranormal [100]. Suzuki et al. reported a transient progression of diabetes from weight gain to increased liver enzyme levels and onward to hypertriglyceridemia (HTG) and then glucose intolerance [101] (Figure 3). 

### 3.4. Nervous System Disease

Metabolic disorders can also cause NSDs, of which stroke [101] and depression [102] are the two most common. Stroke is characterized by blocked blood vessels. Clots form in the brain and cut off blood flow, blocking arteries and causing blood vessels to rupture, leading to bleeding. During a stroke, an artery in the brain ruptures, causing brain cells to suddenly die from lack of oxygen [103]. In one study, metabolism equivalents were associated with a higher risk of recurrent stroke in patients with ischemic stroke [104]. Abdominal obesity, high blood pressure, low HDL-C, and high TG and IR are recognized as risk factors for stroke [104]. Among them, obesity is an important metabolic factor affecting stroke, which leads to a prethrombotic state of inflammation that accelerates the progression of AS [105]. Depression is a metabolic brain disease and a global health challenge [106]. It has been reported that obesity is associated with depression, and the risk of depression is greater when obesity is associated with poor metabolic conditions, including hypertension, dyslipidemia, and high CPR or IR [107]. In addition, a poor diet is also a risk factor for depression [108]. Therefore, these risk factors can trigger a range of responses, such as lethargy, fatigue, excessive sleepiness, binge eating, weight gain, diurnal mood changes, and impaired cognitive performance [109] (Figure 3).

### 3.5. Nonalcoholic Steatohepatitis

NASH is a condition of chronic liver injury and inflammation caused by excess lipid accumulation in the liver [110], and is characterized by hepatocellular damage, inflammation, and fibrosis [111,112]. The pathology of NASH includes steatosis, lobular mixed cell inflammation, hepatocyte degeneration or cell death, and fibrosis [113]. Emerging evidence indicates that NASH is closely associated with obesity, diabetes, and MetS [114]. Among these comorbidities, T2DM appears to be the most important risk factor for NASH and the most important clinical predictor of adverse clinical outcomes [115]. Epidemiological studies have shown that approximately 83% of NASH patients present with hyperlipidemia, 82% of NASH patients are obese, and 48% of NASH patients are diagnosed with T2DM [116,117]. Over time, NASH can progress to cirrhosis, end-stage liver disease, or the need for liver transplantation, and is associated with liver specificity and increased overall mortality. Therefore, early diagnosis and targeted treatment are needed to improve NASH patient outcomes [118] (Figure 3).

### 3.6. Other Metabolic Diseases

In addition, metabolic diseases also include cancer [119], gout [45], and osteoporosis [120]. MetS is reported to be closely associated with cancer because it increases cancer risk and cancer-related mortality [119]. Cancer patients with MetS are reported to have higher mortality rates than patients without MetS [121]. In addition, Mets is associated with an increased risk of several common cancers, including pancreatic, colorectal, liver, endometrial, and postmenopausal breast cancer, in adults [122]. Central obesity and hypercholesterolemia are the main factors leading to the association between MetS and cancer [123,124]. The pathophysiological mechanisms that contribute to MetS cancer development include chronic hyperglycemia, exposure to endocrine disruptors, hyperuricemia and IR, abnormal sex hormone metabolism and adipokines, air pollution, and endocrine changes associated with nightshift work [119]. Gout is a chronic disease of deposition of monosodium urate crystals, which form in the presence of increased urate concentrations [86]. Hyperuricemia is the central risk factor for the development of gout [125]. In addition, a large number of studies have shown that gout is associated with an increased risk of death, mainly due to CVD [107]. Osteoporosis is a bone disease characterized by impaired bone strength that leads to an increased risk of fracture [126], and the risk factors contributing to osteoporosis include smoking, nutrition, neuromuscular function, bone mass, bone size, structure, microstructure and intrinsic properties [127]. In addition, the incidence of osteoporosis increases with age and is associated with higher rates of disability and mortality [127] (Figure 3).

## 4. The Modulation of Plant Polysaccharides in Metabolic Disease

### 4.1. Aloe vera Polysaccharides

*Aloe vera*, belonging to the Liliaceae family, is a short-stemmed plant that stores water in its plants. Phytochemical studies have shown that *Aloe vera* contains polysaccharides, sugars, vitamins, minerals, amino acids, enzymes, sugars, and anthraquinones. APs, being the most abundant compounds, are required for skin care, health care, antioxidative qualities, and wound healing [128]. In addition, APs are reported to have therapeutic potential in metabolic diseases (Table 2). For example, APs protected against cerebral ischemia–reperfusion injury in the middle cerebral artery occlusion (MCAO) of male Wister rats via downregulating the expression of caspase-3, thereby inhibiting neuronal apoptosis. However, this study has not clarified its mechanism of action associated with anti-apoptosis, and more studies are needed to investigate whether APs alleviate cerebral ischemia–reperfusion injury via other pathways such as antioxidation and anti-inflammation [129]. In addition, APs significantly inhibited the apoptosis of palmitate-induced HIT-T15 cells via alleviating endoplasmic reticulum (ER) stress. Mechanistic studies revealed that APs inhibited the activation of PERK and IRE1 pathways and the production of ROS induced by palmitate. Through comparison, AP mixture with a molecular weight greater than 50 kDa showed the best anti-apoptosis and antioxidation activities. In db/db mice, the oral administration of APs significantly decreased the fasting blood glucose (FBG) levels [7]. Therefore, APs have therapeutic potential for MCAO and T2DM via antioxidation and anti-apoptosis (Table 2).

### 4.2. Angelica Sinensis Polysaccharides

*Angelica sinensis* is an important medicinal herb in China. Its medicinal value has been commonly known for a long time, among which the effects of promoting blood circulation and relieving pain, dredging meridians, and regulating meridians are remarkable [130]. Polysaccharides are a kind of bioactive component in *Angelica sinensis* that exhibits beneficial effects in some metabolic diseases, such as AD and diabetes (Table 2). In an Aβ25-35-induced rat model of AD, ASPs improved spatial learning and memory impairment, regulated the balance of neurotransmitters, inhibited the expression of proinflammatory cytokines, including TNF-α, IL-1β, and TNF-α, inhibited the activity of SOD and catalase (CAT), decreased MDA activity, and inhibited the expression of caspase-3 and the ratio of Bax/Bcl-2. Mechanistic studies revealed that APs activated the BDNF/TrkB/CREB signaling pathway to exert a neuroprotective effect [8]. In addition, in the prediabetic and streptozotocin (STZ)-induced diabetic BALB/c mice, the oral administration of ASPs reduced the FBG, alleviated abnormal fasting serum insulin concentrations, decreased the homeostasis model assessment–IR index and body weight, improved the dyslipidemia conditions, reduced serum total cholesterol (TC) or triglyceride (TG) concentrations, increased hepatic glycogen (HG) and muscle glycogen (MG) concentrations, reduced IR-related serum inflammatory factors IL-6 and TNF-α, and restored the impaired pancreatic/hepatic tissues or adipose tissues. All these data indicate that ASPs exert hypoglycemic and hypolipidemic effects via ameliorating IR [36]. Therefore, ASPs are promising therapeutic drugs for AD and diabetes via antioxidation, anti-inflammation, anti-apoptosis, and improving IR (Table 2).

### 4.3. Pumpkin Polysaccharides

Pumpkin, belonging to the family Cucurbitaceae, is an edible plant and an important TCM. Phytochemical studies revealed that polysaccharides, amino acids, fatty acids, protein, carotene, and vitamins are the important components of pumpkin [131]. Emerging evidence indicates that polysaccharides are the most abundant carbohydrate in pumpkin and have a variety of biological activities, including antibacterial, antidiabetic, anti-inflammatory, antioxidant, and anticancer [131]. Recently, pumpkin polysaccharides (PPs) have been reported to improve metabolic disorder in many diseases (Table 2). For example, PPs remodeled intestinal microbiota by reducing *Erysipelotrichaceae* and increasing the abundance of *Achmania*, thereby reducing FBG, IR and blood lipid TC, TG, and LDL levels, and improving blood glucose and lipid metabolism in T2DM rats [132,133]. This study indicates that PPs have therapeutic potential for T2DM through modulating intestinal microbiota. In rats fed with high-fat diet, PPs reduced the levels of TG, TC, and plasma LDL-C, and increased the levels of fecal fat, cholesterol, and plasma HDL-C. Mechanistic studies found that PPs increased the binding capacity of fat and cholesterol to improve obesity [134]. Therefore, PPs may become an effective drug to treat T2DM and obesity through regulating intestinal microbiota, lipid, and other metabolic pathways (Table 2).

### 4.4. Lycium Barbarum Polysaccharides

*Lycium barbarum* (wolfberry), belonging to the family of Solanaceae, is a shrub native to China. Phytochemical studies have shown that *Lycium barbarum* contains polysaccharides, carotenoids, and polyphenols such as caffeic acid, chlorogenic acid, and p-coumaric acid [135]. Among then, *Lycium barbarum* polysaccharides (LBPs) are the major components and have a variety of medicinal values, including antioxidative, anticancer, antifatigue, and antiaging effects [136]. Recently, LBPs have been reported to exhibit beneficial effects in many metabolic diseases (Table 2). In high-fat diet and STZ-induced diabetic rats, the oral administration of LBPs reduced the concentration of albuminuria, blood urea nitrogen, IL-2, IL-6, TNF-α, IFN-α, serum levels of monocyte chemoattractant protein-1 (MCP-1), and cell adhesion molecule-1 (CAM-1), and increased the activity of SOD and glutathione peroxidase (GSH Px) in serum. Mechanistic studies show that LBPs inhibited inflammation and oxidative stress by inhibiting the NF-κB pathway [137]. In addition, the oral administration of LBPs reduced serum TG, TC, and LDL-C levels, and increased HDL-C levels and the production of short-chain fatty acids (SCFA) in obese mice. These data indicate that LBPs promote lipid metabolism by improving IR and fatty acid oxidation, activating the adenosine monophosphate-activated protein kinase CoA carboxylase pathway [138]. Therefore, LBPs have become potential drugs for treating diabetes and obesity mainly through improving IR, and antioxidation and anti-inflammatory effects (Table 2).

### 4.5. Ginseng Polysaccharides

*Ginseng* has a long history as a medicinal herb for the treatment of human diseases in many Eastern countries, including China, Korea, and Japan. Phytochemical studies have shown that *Ginseng* contains terpenoids, flavonoids, lignans, sterols, and other compounds. To date, polysaccharides have been identified and extensively studied for their pharmacological activities, including immunoregulation, antitumor, antibacterial, anti-inflammatory, and antioxidative effects [139]. In addition, *Ginseng* polysaccharides (GPs) exhibit a regulatory effect on metabolic disorders in many diseases (Table 2). For example, in open-field test-induced anxiety C57BL/6, the oral administration of GPs increased the walking distance and staying time in the central area of the mice and decreased their average speed. Mechanistic studies revealed that GPs reduced the expression of tyrosine hydroxylase (TH) in the midbrain and dopamine D1 receptor (DRD1) [140]. In high glucose diet and STZ-induced rats, the oral administration of GPs reduced the FBG of rats, restored the disturbed intestinal flora, and enhanced the β-production capacity of d-glucosidase, which enhances the hypoglycemic effect of ginsenoside Rdb1. The mechanistic study showed that GPs changed the biotransformation pathway of ginsenoside Rb1 and improved the biotransformation rate of ginsenoside Rb1 to CK [141]. Therefore, GPs may have therapeutic potential for anxiety and diabetes by regulating the center and improving intestinal flora (Table 2).

### 4.6. Schisandra Chinensis Polysaccharides

*Schisandra chinensis*, also known as magnolia berry or five-flavor fruit, is a famous Chinese herbal medicine, with its traditional efficacies of calming nerves, delaying double aging, preventing CVD, and inhibiting fatigue. Phytochemical studies have shown that dibenzocyclooctadiene lignans and triterpenoids are the important components in *Schisandra chinensis*. In addition, polysaccharides have been identified as important bioactive components in *Schisandra chinensis*, which exhibit many biological activities, such as antitumor, immune enhancement, and liver protection [64]. In addition, *Schisandra chinensis* polysaccharides (SCPs) improve metabolic diseases via regulating metabolism pathways (Table 2). In chronic fatigue syndrome (CFS) rats induced by restraint stress, forced exercise, and crowded noisy environment, the oral administration of SCPs significantly increased the daily food intake, weight, spatial memory, escape ability, and staying time in water of mice. Mechanistic studies revealed that SCPs promoted the recovery of the tricarboxylic acid cycle metabolism pathway and the alanine, aspartic acid, and glutamate metabolism pathways [142]. In STZ-induced T2DM rats, SCPs reduced FBG, increased fasting insulin level, improved glucose tolerance, and inhibited the expression of proinflammatory cytokines, including TNF-α and IL-1β. Mechanistic studies revealed that SCPs downregulated the NF-κ B and P-JNK signaling pathways and upregulated the IRS-1/PI3K/AKT signaling pathway [143]. In HFD-induced male NAFLD rats, SCPs reduced the serum level of AST, ALT, TG, TC, and LDL-C, and increased the level of HDL-C, indicating that SCPs alleviate the occurrence of NAFLD by regulating the expression of UDP-glucose pyrophosphorylase (UGP2), UDP-glucose 6-dehydrogenase (UGDH), acetyl coenzyme carboxylase (ACC), and fatty acid synthase (FAS) in the liver of NAFLD rats [144]. Therefore, SCPs can effectively improve CFS, diabetes, and NAFLD by regulating metabolism pathways and inhibiting inflammatory response (Table 2).

### 4.7. Dioscorea Opposita Polysaccharides

*Dioscorea opposita*, also known as Chinese Yam, is an edible and medicinal tuber crop in China, indicating low toxicity and high safety for humans. It is widely used to treat diabetes, diarrhea, asthma, and other diseases. Modern phytochemistry studies have shown that *Dioscorea opposite* contains polysaccharides, amino acids, fatty acids, and steroids. Among then, *Dioscorea opposite* polysaccharides (DOPs) are one of the main bioactive substances that exhibit many important biological activities, such as hypoglycemic, immunomodulatory, antioxidative, and antitumor activities [145]. In addition, DOPs exhibit an improvement effect in metabolic diseases (Table 2). For example, in alloxan-induced diabetes mellitus rats, DOPs reduced blood glucose, increased insulin secretion, and improved the function of pancreatic β-cells. Its mechanism is closely associated with a reduction in lipid peroxide and the effective elimination of free radicals, leading to the amelioration of tissue damage and the promotion of tissue repair and regeneration [146]. In dexamethasone-induced IR glucose/lipid metabolism diabetic mice, DOPs reduced blood glucose via promoting the repair of β-insulin cells [147]. Therefore, DOPs as a healthy functional food have therapeutic potential for diabetes (Table 2).

### 4.8. Poria Cocos Polysaccharides

*Poria cocos*, belonging to the fungus family of Polyporaceae, is an edible fungus. In addition, *Poria cocos* has been used as a TCM for more than 2000 years. Phytochemical studies have shown that polysaccharides, triterpenes, sterols, amino acids, fatty acids, etc., are the major components in *Poria cocos*. Among them, PCPs have a wide range of biological activities, including antidiabetic, antitumor, immunoregulation, anti-inflammatory, antioxidation, and antiaging effects [27]. In high-fat diet-induced NAFLD mice, the oral administration of PCPs decreased serum and hepatic lipid levels, increased lipid utilization, and decreased lipid synthesis and absorption. Its mechanism is closely associated with regulation of fatty acid metabolism, bile acid metabolism, and tricarboxylic acid cycle [148]. In addition, the oral administration of PCPs reduced serum TNF-α, IL-6, NO, LDLC, TG, and TC levels in high-fat diet-induced Apoe−/− mice, Meanwhile, PCPs exerts antioxidative effect via decreasing the malondialdehyde (MDA) concentration and increased the activity of SOD. Mechanistic studies revealed that PCPs inhibited the TLR4/NF-κB pathway to reduce inflammatory factors and blood lipid levels [9]. Therefore, PCPs improve metabolic diseases such as NAFLD and AS by improving metabolism pathways and inhibiting inflammatory response and oxidative stress (Table 2).

### 4.9. Tea (Camellia sinensis) Polysaccharides

Tea is a nonalcoholic drink containing polyphenols such as catechin, epicatechin, epicatechin gallate, gallocatechin, and epigallocatechin. In addition, tea polysaccharides (TPs) are also recognized as the main bioactive components. Increasing studies have shown that TPs have various biological activities, including antioxidative, antitumor, hypoglycemic, and hypolipidemic effects [79]. In STZ-induced T2DM rats, the oral administration of acidic TPs significantly improved plasma and liver lipid metabolism and changed the composition of intestinal flora, as evidenced by decreased *Bifidobacterium, Blautia, Dorea, Oscillospira, Desulfovibrio*, and *Lactobacillus* species. Mechanistic studies revealed that TPs regulated the primary and secondary bile acid biosynthesis and downregulated the NOD-like receptor signaling pathway, lipopolysaccharide biosynthesis, and the insulin signaling pathway [149]. In the formalin test and several behavioral animal models, the oral administration of TPs dose-dependently decreased the number of crossings in the activity chamber and in the open field test, and reduced the number of buried marbles. These results suggest that TPs exert antinociceptive, sedative, and anxiolytic-like effects. However, whether the mechanism of action of TPs is associated with the interference of CNS is unknown, and needs more future studies to elucidate [150]. Therefore, TPs may become effective drugs to treat T2DM and CNS-related pain and anxiety through regulating the metabolism pathways and gut microbiota (Table 2).

**Table 2:** The pharmacological activities and action mechanisms of PPS in the models of various metabolic diseases.

PPS	Dosage	Model	Effect	Mechanism	Diseases
APs	60 mg/kg	MCAO male Wister rats (in vivo)	Regulating immunity, resisting tumor, protecting liver, and nourishing stomach	Inhibiting aoptosis	Cerebral ischemia [129]
	5, 10 and 20 mg/ml 100 mg/g	Palmitate-induced HIT-T15 cells (in vitro) db/db mice (in vivo)	Regulating ER stress, inhibiting neuronal apoptosis, reducing blood sugar	Inhibiting PERK and IRE1 pathways, inhibiting ROS generation	T2DM [7]
ASPs	50 mg/kg	Hippocampus was injected with Aß25 - 35 rats (in vivo)	Inhibiting inflammation and apoptosis	Activating the BDNF/TrkB/CREB pathway	AD [8]
	400 and 600 mg/kg	STZ-induced diabetic BALB/c mice (in vivo)	Inhibiting TNF-α, IL-1β, and TNF-α expression, inhibiting SOD and CAT activity, decreasing MDA content, inhibiting caspase-3 and Bax/Bcl-2 expression	Activating the BDNF/TrkB/CREB signaling pathway	T2DM [35]
PPs	100, 250 and 500 mg/kg	STZ-induced rats (in vivo)	Reducing FBG, IR and blood lipid TC, TG and LDL levels, improving blood glucose		T2DM [133]
	95% (*w/w*) HF diet plus 5% (*w/w*) PP	Male Sprague Dawley rats (in vivo)	Reducing TG, TC, and plasma LDL-C, increasing the levels of fecal fat, cholesterol, and plasma HDL-C	Increasing the binding capacity of fat and cholesterol	Obesity [134]
LBPs	100, 250, and 500 mg/kg	STZ induced diabetic rat (in vivo)	Reducing the concentration of albuminuria, blood urea nitrogen, IL-2, IL-6, TNF-α, IFN-α, serum levels of MCP-1 and ICAM-1, increasing SOD and GSH Px activity	Inhibiting the NF-κB pathway	T2DM [137]
	0.2% LBPs water	HFD mice (in vivo)	Reducing TG, TC and LDL-C levels, increasing HDL-C and SCFA	Improving IR and fatty acid oxidation, activating the adenosine monophosphate activated protein kinase CoA carboxylase pathway	Obesity [138]
GPs	50 and 200 mg/kg	C57BL/6 anxiety mice (in vivo)	Increasing the walking distance and staying time in the central area of the mice, decreasing the average speed of mice	Reducing the expression of tyrosine hydroxylase (TH) in the midbrain and dopamine D1 receptor (DRD1)	Anxiety [140]
	0.2, 0.5 and 1 g/kg	High-sugar diet and STZ -induced rats (in vivo)	Reducing FBG, restoring disturbed intestinal flora, enhancing β- d-glucosidase, enhancing the hypoglycemic effect of ginsenoside Rdb1	Changing the biotransformation pathway of ginsenoside Rb1, improving the biotransformation rate of ginsenoside Rb1 to CK	T2DM [141]
SCPs	200 mg/kg	CFS rats (in vivo)	Increasing food intake and body weight, improving the memory deficit	Promoting the recovery of tricarboxylic acid cycle metabolism pathway and alanine, aspartic acid and glutamate metabolism pathway	CFS [142]
	25, 50 or 100 mg/kg	STZ -induced rats (in vivo)	Reducing FBG, increasing fasting insulin level, improving glucose tolerance, and inhibiting the expression of proinflammatory cytokines	Downregulating NF- κ B and P-JNK signaling pathways, upregulating the IRS-1/PI3K/AKT signaling pathway	T2DM [143]
	100 mg kg	High-fat diet-induced male Wistar rats (in vivo)	Reducing AST, ALT, TG, TC, and LDL-C, increasing HDL-C	Regulating UGP2, UGDH, ACC and FAS expression	NASH [144]
OPs		alloxan diabetic rats (in vivo)	Reducing blood glucose, increasing insulin secretion, and improving the function of pancreatic β-cells	Reducing lipid peroxide and eliminating free radicals	Diabetes [146]
	50, 100 and 150 mg/kg	Dexamethasone-induced IR glucose/lipid metabolism diabetic mice)(in vivo)	Reducing blood sugar		T2DM [147]
PCPs	100, 200, and 400 mg/kg	ApoE−/− mice (in vivo)	Reduced serum TNF-α, IL-6, NO, LDL-C, TG and TC levels, decreasing MDA, and increasing SOD	Inhibiting the TLR4/NF-κB pathway	AS [9]
	3 g/day	High-fat diet-induced NAFLD mice (in vivo)	Increasing the lipid utilization, decreasing the lipid synthesis and absorption	Regulating fatty acid metabolism, bile acid metabolism, and tricarboxylic acid cycle	NAFLD [148]
TPs	200, 400 and 800 mg/kg	STZ-induced T2DM rats (in vivo)	Reducing intestinal flora	Regulating primary and secondary bile acid biosynthesis, downregulating the OD-like receptor signaling pathway	T2DM [149]
	3, 10, and 30 mg/kg	Formalin test and several behavioral animal models (in vivo)	Resisting anxiety, pain, anxiety		Anxiety [150]

## 5. Conclusions and Perspective

**Figure 4:**
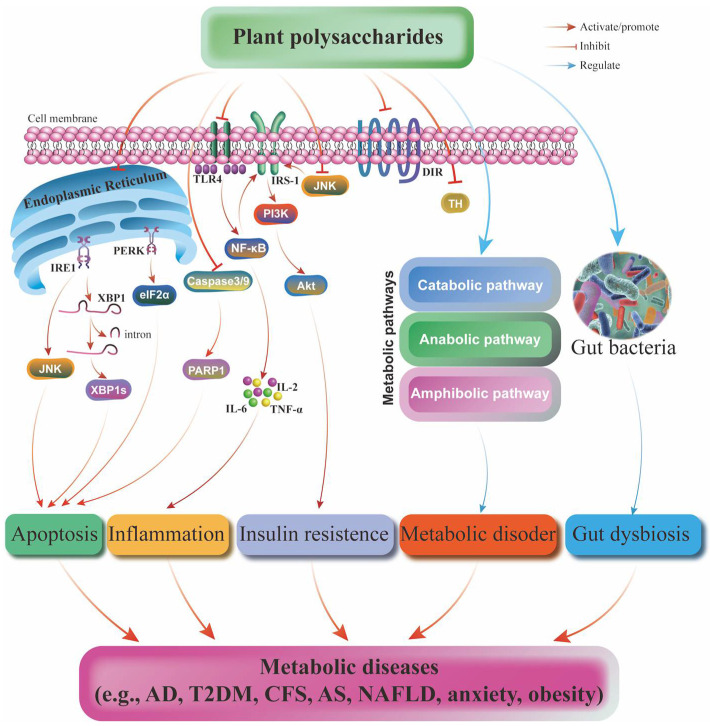
The mechanism of action of PPS in various metabolic diseases, including AD, T2DM, CFS, AS, NAFLD, anxiety, and obesity. The mechanism of action mainly involves the apoptotic, inflammatory and IR pathways and the regulation of metabolic pathways and gut bacteria.

Metabolic diseases, including obesity, diabetes, CVD, NASH, NSDs, and cancer, are characterized by the disorder of the generation and storage of energy. They can be affected by the common risks from genetics, epigenetics, susceptibility, environmental factors, and nutrition. PPS are an important class of biopolymers with a wide range of sources and varieties. They contain more than 10 monosaccharides linked by glycosidic bonds. Most PPS from edible plants have high safety and exhibit beneficial effects in many metabolic diseases such as cerebral ischemia, T2DM, AD, CFS, NASH, AS, and NAFLD. Their mechanisms of action are associated with the regulation of apoptotic, inflammatory, oxidative stress, gut microbiota, and many metabolic pathways (Figure 4). As a natural product, PPS allow them to be used as substitutes for fat or sugar. Therefore, the clinical value and broad application prospects of PPS can allow them to be developed into a series of functional foods in the future. However, there are still many problems to be solved. First, although the development of GC-MS, X-ray fiber diffraction, mass spectrometry, nuclear magnetic resonance, electron diffraction, and other analytical techniques makes it possible to obtain some structural information of PPS, there are still many difficulties and challenges in the elucidation of the complicated structure of polysaccharides. In addition, the unelucidated or unambiguous structure makes a structure-activity study difficult. In recent years, the research mainly focused on the effects and mechanisms of PPS on obesity and T2DM. In future, more attention should be paid to exploring the therapeutic potential and mechanism of action of other metabolic diseases, such as osteoporosis, hyperuricemia, and other diseases. Finally, this review should provide directions and references for the future study of PPS in metabolic diseases.

## Data Availability

Data sharing not applicable.
